# Editorial: Plant-microbe interactions for agricultural sustainability facing environmental challenge

**DOI:** 10.3389/fpls.2023.1310616

**Published:** 2023-10-24

**Authors:** Naeem Khan, Mohamed Lazali

**Affiliations:** ^1^ Department of Agronomy, University of Florida, Gainesville, FL, United States; ^2^ Plant Production Laboratory, University of Khemis Miliana, Tlemsin, Algeria; ^3^ Laboratoire de recherche ERP, Faculté des Sciences de la Nature et de la Vie & des Sciences de la Terre, Université Djilali Bounaama de Khemis Miliana, Khemis Miliana, Algeria

**Keywords:** plant microbe interaction, agriculture sustainability, plant metabolites, abiotic stresses, stress responsive genes

In the face of escalating environmental stresses affecting plant productivity globally, including biotic and abiotic challenges, sustainable agriculture practices are crucial. Pesticide and chemical fertilizer use degrade soil fertility and pollutes the environment, necessitating eco-friendly agricultural solutions. Soil microorganisms emerge as vital agents in plant protection during stress, enhancing growth through various mechanisms, such as phytohormone regulation and nutrient uptake. These microorganisms also bolster plant tolerance to biotic stresses, acting through induced systemic resistance mechanisms. Amid climate change-induced challenges like flooding, drought, and extreme temperatures, crop yields are severely impacted. Water scarcity, heatwaves, and severe droughts have led to reduced soil moisture levels and altered plant physiology. Additionally, dust pollution poses a significant environmental concern, emphasizing the need for green plant development in urban areas to counter air pollution. Climate change’s impact on plant-pathogen interactions is multifaceted, potentially altering pathogen biology, host development, and disease severity. These modifications can either exacerbate or mitigate disease incidence. The evolving climate affects soil pathogens directly and indirectly, altering their abundance and influencing plant-mediated soil carbon inputs. As global climate change persists, understanding these complex interactions is vital for developing effective strategies to ensure sustainable agriculture in the future.

Flooding has a detrimental impact on wheat plants, leading to reduced root biomass during tillering and significant decreases in shoot and root dry biomass at the booting stage. Flooded wheat plants also exhibited decreased numbers of tillers and spikes. Soil properties were significantly affected by flooding, with increased soil moisture, pH, zinc, and available phosphorus observed in flooded soil samples. Flooded plants experienced lower concentrations of essential nutrients such as nitrogen, sulfur, phosphorus, magnesium, and potassium in both roots and leaves, especially in the early stages of growth. The study also found significant differences in the fungal community among various plant compartments. The interaction between plant growth stage and flooding also played a significant role in shaping the wheat mycobiota. Principal coordinates analysis distinguished samples associated with specific plant growth stages and separated flooded samples from their respective controls. Root carbon and manganese, along with leaf nitrogen, magnesium, and manganese content, significantly influenced root-associated fungal assembly. Rhizosphere fungal community composition was primarily affected by soil parameters like pH, soil potassium, and plant dry leaf weight, as well as root nitrogen and sodium concentration. In contrast, leaf mycobiota was significantly influenced by leaf traits such as sodium, magnesium, manganese, and potassium concentration, as well as root potassium and manganese concentration (Francioli et al.).

In the face of climate change-induced drought stress leading to a significant drop in global rice production and threatening food security, this study focused on isolating and characterizing eight drought-tolerant bacteria from rice rhizosphere capable of withstanding 20% PEG-8000. These strains displayed various plant growth-promoting traits, including ACC deaminase activity, exopolysaccharide production, phosphate-solubilizing activity, indole-3 acetic acid production, and organic acid production. A bacterial consortium comprising *Bacillus subtilis* NM-2, *Brucella haematophilum* NM-4, and *Bacillus cereus* NM-6 was found to markedly enhance seedling growth and vigor index compared to non-inoculated stressed plants. Rhizoscanning confirmed improved root parameters such as length, diameter, and surface area across tested genotypes. The consortium’s positive impact correlated with enhanced plant growth and stress tolerance traits, such as increased proline content, relative water content, membrane stability index, and production of antioxidant enzymes. Additionally, infrared thermal imaging and soil plant analyzer development revealed reduced temperature and improved chlorophyll content in inoculated plants. Inoculation significantly enhanced photosynthetic parameters like reference CO_2_, photosynthetically active radiation, sub-stomatal CO_2_, transpiration rate, and photosynthetic rate, particularly in stressed conditions. Principal component analysis highlighted the consortium’s contribution to stress responses and yield in tested rice genotypes, offering a promising solution to mitigate water scarcity-related challenges in the context of evolving climate uncertainties (Mahreen et al.).

In another study Zilaie et al. reported the impact of dust stress on three desert plant species (*Seidlitzia rosmarinus, Haloxylon aphyllum*, and *Nitraria schoberi*) and the potential of plant growth-promoting bacterial strains (*Zhihengliuella halotolerans SB* and *Bacillus pumilus* HR) to enhance their Air Pollution Tolerance Index (APTI) were investigated. Dust exposure led to significant reductions in chlorophyll content, leaf relative water content, APTI, and protein content in the plants. However, the application of *Z. halotolerans* SB increased chlorophyll content in *H. aphyllum* and *S. rosmarinus* and enhanced ascorbic acid levels in H*. aphyllum* and *N. schoberi*. *B. pumilus* HR improved leaf relative water content in *H. aphyllum* and *N. schoberi* and increased protein concentration in all three plants. Both bacterial strains also reduced peroxidase activity in the plants. Under dust stress, *H. aphyllum* exhibited a higher APTI than the other species, and *Z. halotolerans* SB was more effective for *S. rosmarinus*. The study concluded that these plant growth-promoting rhizobacteria effectively enhanced plant tolerance to air pollution, showcasing their potential in green belt development. Additionally, dust treatments without bacterial intervention caused increased levels of ascorbic acid in the plants, with the impact varying across species. Notably, bacterial inoculation significantly reduced leaf pH in all three species, particularly in *N. schoberi*. These findings underscore the importance of beneficial bacteria in mitigating the adverse effects of dust stress on plants and highlight species-specific responses to both dust and bacterial interventions.

Sbeiti et al. explored the impact of increasing temperatures on disease resistance to *Verticillium* spp., a soil-borne fungal pathogen, in *Medicago truncatula* and *Medicago sativa*. Twelve pathogenic strains were analyzed for their growth and pathogenicity at different temperatures. A strain of *V. alfalfae* was evolved at 28°C, making it more aggressive than the wild type at this temperature. When inoculated on different *M. truncatula* genotypes and alfalfa varieties at 20°C, 25°C, and 28°C, some lines shifted from resistant to tolerant or from partially resistant to susceptible with rising temperatures. Susceptible lines exhibited reduced plant fitness due to disease. The study revealed that root-pathogen interactions are affected by global warming, leading to increased plant susceptibility and greater virulence in hot-adapted strains. However, among the tested strains, none appeared to be truly adapted to higher temperatures, indicating potential challenges in studying plant responses under future climate change scenarios.

In another study, plant-microbe interactions were investigated, focusing on the roles of SlWRKY, SlGRAS, and SlERF genes during the symbiotic association of *Curvularia lunata* SL1 with tomato plants. RNA-seq data were analyzed, revealing that more than half of the studied SlWRKY genes, including SlWRKY38, SlWRKY46, SlWRKY19, and SlWRKY51, were significantly upregulated during the symbiotic association. Several SlGRAS and SlERF genes, such as SlGLD2, SlGLD1, SlERF.C.5, ERF16, and SlERF.B12, were also upregulated. Additionally, the study explored the potential roles of these genes in hormonal regulation during plant-microbe interactions, identifying upregulated transcripts associated with plant hormone signaling pathways. To validate the RNA-seq data, RT-qPCR analyses of selected genes were performed, confirming the accuracy of the expression patterns observed. The study provided new insights into the differential expression profiles of these genes during symbiotic associations, shedding light on their potential roles in hormonal regulation. Furthermore, phylogenetic analyses were conducted, classifying SlWRKY, SlGRAS, and SlERF TFs into distinct clades, providing a comprehensive understanding of the evolutionary relationships among these gene families in Solanum lycopersicum and *Arabidopsis thaliana* (Khan et al.).

In response to the rising number of slopes due to societal and economic growth, addressing slope instability through ecological protection and reconstruction has become a global concern. A current study investigated eight strategies (A, B, C, AB, AC, BC, ABC, CK) involving specific mineral solubilizing microorganisms (MSMs) for active permanent greening (APG) on slopes. The findings demonstrated that MSMs significantly improved soil quality by increasing effective metal ions, available nutrients, and soil enzyme activity. Among the strategies, strategy A outperformed others, enhancing soil components like calcium, magnesium, potassium, nitrogen, phosphorus, and organic matter by substantial percentages. Urease, sucrase, and peroxidase activities were also notably boosted. Amorpha growth was significantly promoted, especially with strategy A, showing substantial increases in seedling height, ground diameter, leaf area, root length, and root volume. A comprehensive evaluation using entropy-analysis hierarchy process highlighted strategy A as the most promising. Field tests confirmed that APG outperformed traditional greening methods, ensuring high vegetation cover and stable soil structure. The study’s results serve as a practical foundation and technical guide for the development and application of APG. The analysis ranked different strategies based on their impact on soil, plants, roots, and the overall system, with strategy A emerging as the most effective approach (Wang et al.).

## Author contributions

NK: Conceptualization, Formal Analysis, Funding acquisition, Project administration, Resources, Software, Supervision, Writing – original draft, Writing – review & editing. ML: Formal Analysis, Project administration, Writing – review & editing.

